# Oxytocin‐monomeric red fluorescent protein 1 synthesis in the hypothalamus under osmotic challenge and acute hypovolemia in a transgenic rat line

**DOI:** 10.14814/phy2.14558

**Published:** 2020-09-10

**Authors:** Hiromichi Ueno, Kenya Sanada, Tetsu Miyamoto, Kazuhiko Baba, Kentaro Tanaka, Haruki Nishimura, Kazuaki Nishimura, Satomi Sonoda, Mitsuhiro Yoshimura, Takashi Maruyama, Yasushi Oginosawa, Masaru Araki, Shinjo Sonoda, Tatsushi Onaka, Yutaka Otsuji, Yoichi Ueta

**Affiliations:** ^1^ Department of the Second Department of Internal Medicine University of Occupational and Environmental Health Kitakyushu Japan; ^2^ Physiology School of Medicine University of Occupational and Environmental Health Kitakyushu Japan; ^3^ Division of Brain and Neurophysiology Department of Physiology Jichi Medical University Shimotsuke Japan

**Keywords:** hyperosmolality, hypothalamus, hypovolemia, oxytocin, transgenic rat

## Abstract

We generated a transgenic rat line that expresses oxytocin (OXT)‐monomeric red fluorescent protein 1 (mRFP1) fusion gene to visualize the dynamics of OXT. In this transgenic rat line, hypothalamic OXT can be assessed under diverse physiological and pathophysiological conditions by semiquantitative fluorometry of mRFP1 fluorescence intensity as a surrogate marker for endogenous OXT. Using this transgenic rat line, we identified the changes in hypothalamic OXT synthesis under various physiological conditions. However, few reports have directly examined hypothalamic OXT synthesis under hyperosmolality or hypovolemia. In this study, hypothalamic OXT synthesis was investigated using the transgenic rat line after acute osmotic challenge and acute hypovolemia induced by intraperitoneal (i.p.) administration of 3% hypertonic saline (HTN) and polyethylene glycol (PEG), respectively. The mRFP1 fluorescence intensity in the paraventricular (PVN) and supraoptic nuclei (SON) was significantly increased after i.p. administration of HTN and PEG, along with robust Fos‐like immunoreactivity (co‐expression). Fos expression showed neuronal activation in the brain regions that are associated with the hypothalamus and/or are involved in maintaining water and electrolyte homeostasis in HTN‐ and PEG‐treated rats. *OXT* and *mRFP1* gene expressions were dramatically increased after HTN and PEG administration. The plasma OXT level was extremely increased after HTN and PEG administration. Acute osmotic challenge and acute hypovolemia induced upregulation of hypothalamic OXT in the PVN and SON. These results suggest that not only endogenous arginine vasopressin (AVP) but also endogenous OXT has a key role in maintaining body fluid homeostasis to cope with hyperosmolality and hypovolemia.

## Graphical abstract

CVOs activation under hyperosmolality, and brainstem neurons and SFO activation under hypovolemia could upregulate OXT synthesis in the hypothalamus.

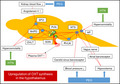

## 1 INTRODUCTION

Oxytocin (OXT) is a neurohormone with a structure similar to that of arginine vasopressin (AVP), and both hormones are synthesized mainly in the paraventricular nucleus (PVN) and supraoptic nucleus (SON) of the hypothalamus. The two hormones, AVP and OXT, diverged during evolution from a common ancestor called vasotocin. The PVN is divided into two areas, and OXT synthesized in the magnocellular division in the PVN (mPVN) is carried to the posterior pituitary (PP) by axonal transport and released into the bloodstream Sofroniew, 1980. On the other hand, the axons of OXT‐expressing neurons in the dorsal parvocellular division of the PVN (dpPVN) project to the central nervous system and the autonomic nervous system in the brainstem area. AVP is known to be an integral hormone in maintaining water and electrolyte homeostasis by regulating the reabsorption of water in the collecting ducts of the kidney. AVP plays an important role in social behavior and handling of stress. On the other hand, OXT has various physiological actions, such as uterine contraction, handling of stress, and pain alleviation (Arase et al., 2018; Bernal, Mahía, Mediavilla, & Puerto, 2015; Meyer‐Lindenberg, Leng, Selvarajah, Bicknell, & Russell, 2011). Furthermore, promotion of natriuresis, stimulation of secretion of atrial natriuretic peptide, and suppression of salt intake have been reported as physiological actions of OXT (Blackburn, Samson, Fulton, Stricker, & Verbalis, 1993; Forsling, Brennan, Nelson, & Robertson, 1994; Gutkowska, Shiromani, Jirikowski, & Bloom, 1997; Miyata, Domes, Kirsch, & Heinrichs, 2001; Walter et al., 2000). Given these, OXT and AVP have many similar physiological effects. Hypothalamic AVP synthesis is known to be upregulated mainly under hyperosmolality and hypovolemia (Arima et al., 1999; Dunn et al., 1973; Kondo et al., 2004). The vascular organ of the lamina terminalis (OVLT) and the subfornical organ (SFO), brain regions known as the circumventricular organs (CVOs), sense the change in plasma osmolality (McKinley et al., 2004; Dos santos moreira et al., 2017). The anatomical feature of lacking a blood–brain barrier enables CVOs to directly sense the change in plasma osmolality. The plasma osmolality change sensed by the OVLT and SFO is mainly transmitted to the PVN and SON via the median preoptic nucleus (MnPO), affecting AVP synthesis in the hypothalamus (Antunes‐Rodrigues et al., 2013; McKinley & Johnson, 2004). On the other hand, hypovolemia and the accompanying decrease in blood pressure are detected by baroreceptors in the carotid sinus, aortic arch, right atrium, and pulmonary veins. The decrease in circulating blood volume sensed by baroreceptors is input to the solitary nucleus (NTS) of the brainstem via the vagus and glossopharyngeal nerves. The neuronal signals then activate the brainstem sympathetic nerves including the rostral ventrolateral medulla (RVLM) and upregulate AVP synthesis in the PVN and SON (Berecek & Swords, 1990; Bisset & Chowdrey, 1988; Cruz, Bonagamba, Machado, Biancardi, & Stern, 2008). If OXT has many AVP‐like characteristics, we hypothesized that acute changes in osmolality and circulating blood volume could have a significant effect on hypothalamic OXT synthesis, similar to hypothalamic AVP synthesis. In fact, previous reports have shown elevated plasma OXT levels and activation of hypothalamic OXT neurons under hypovolemia and osmotic challenge (Giovannelli et al., 1990; Landgraf et al., 1988; Shibuki et al., 1988). However, few studies have directly examined hypothalamic OXT synthesis under hyperosmolality or hypovolemia. In addition, quantitative and time‐course analyses of hypothalamic OXT synthesis have not been performed under these conditions.

We generated transgenic rats expressing OXT‐monomeric red fluorescent protein 1 (mRFP1) fusion gene to visualize the dynamics of OXT in the central nervous system (Katoh, Coleman, Doheny, & Travagli, 2011). The OXT‐mRFP1 fluorescence intensity has been reported to closely parallel that for endogenous OXT synthesis. Thus, transgenic rats enable us to semiquantitatively evaluate the production of OXT in local brain regions, such as the PVN and SON, by observing mRFP1 fluorescence intensity, used as a surrogate reporter, with an image analyzer. Using these characteristics of OXT‐mRFP1 transgenic rats, we have shown the changes in hypothalamic OXT synthesis under various physiological conditions (Arase et al., 2018; Matsuura et al., 2016; Motojima, Cohen, Hempstead, & Curran, 2016; Ohno et al., 2018).

In the present experiment, visualization and semiquantitative evaluation of hypothalamic OXT synthesis were attempted under osmotic challenge and acute hypovolemia using the transgenic rat line. Peripheral administration of hypertonic saline (HTN) is a representative experimental approach to create an osmotic challenge. On the other hand, polyethylene glycol (PEG) administration is a representative experimental approach to cause acute hypovolemia. First, we verified the changes in OXT‐mRFP1 by measuring the mRFP1 fluorescence intensity, which is a useful reporter for OXT synthesis, under osmotic challenge and acute hypovolemia in the transgenic rats. Second, we labeled Fos protein, which is an indicator of neuronal activity, with green fluorescence using fluorescent immunohistochemical staining (Fos‐IR), to evaluate the neuronal activity of hypothalamic OXT neurons under osmotic challenge and acute hypovolemia. Third, we examined Fos‐IR cells in the OVLT, MnPO, SFO, area postrema (AP), NTS, and RVLM, which are known to be brain regions that are neurally connected to OXT neurons in the hypothalamus and/or are involved in sensing water and electrolyte changes, under osmotic challenge and acute hypovolemia. Next, we evaluated the gene expressions of *mRFP1* (*mRFP1* mRNA) and *OXT* (*OXT* mRNA) in the hypothalamus in the HTN‐ and PEG‐treated rats. Finally, we examined the plasma OXT levels under osmotic challenge and acute hypovolemia.

## 2 MATERIALS AND METHODS

### 2.1 Experimental animals

Adult male OXT‐mRFP1 rats identified by polymerase chain reaction (7 weeks old, 200–320 mg/body weight, Wistar‐based animals) were used for this experiment (Arase et al., 2018; Katoh et al., 2011). They were housed three per cage under standard conditions at 23–25°C with a 12‐hr light‐dark cycle (light on at 7:15). All rats were treated with daily handling by the authors to relieve the experimental stress a week before the experiment. All experimental methods were approved by the Animal Ethics Committee of the University of Occupational and Environmental Health (Kitakyushu, Japan) and were performed strictly following the guidelines on the use and management of experimental animals of the Japan Physiological Society.

### 2.2 Test substances

PEG (Molecular Weight 4000, Nacalai Tesque, Kyoto, Japan) was prepared by dissolving it in 0.9% saline (20% w/v). HTN was prepared by dissolving 3% NaCl in chemically pure water.

### 2.3 Experimental procedure

All OXT‐mRFP1 rats were randomly separated into four groups: the untreated group, the intraperitoneal (i.p.) administration of saline group, the HTN group, and the PEG group. All rats could consume water and food until 24 hr before the experiment (water: tap water, food: CLEA Rodent Diet CE‐2, CLEA Japan, Inc., Tokyo, Japan). All rats, except the untreated group, were water‐deprived 24 hr before the experiment, without food deprivation. There were no dietary restrictions in this experiment.

### 2.4 Hypothalamic OXT‐mRFP1 fluorescence intensity under osmotic challenge and acute hypovolemia

To measure the changes in OXT‐mRFP1, transgenic rats were sacrificed by transcardial perfusion at 0, 3, 6, 12, and 24 hr after administration of saline, HTN, or PEG at 10:00–12:00 (*n* = 6 in each group). All rats were deeply sedated after administration of the anesthetic mixture, which consisted of medetomidine (3 mg/100 g), midazolam (4 mg/100 g), and butorphanol (5 mg/100 kg), before perfusion (Kawai et al., 2011; Ueno et al., 2019). Transcardial perfusion was performed as described previously (Ueno et al., 2018, 2019). After thoracotomy, the heart was carefully exposed. Perfusion was performed by administering 0.1 M phosphate buffer (PB, pH 7.4), dissolved heparin (1 U/mL), and then 4% (w/v) paraformaldehyde in 0.1 M PB (4% PHA) from the left ventricular apex. Rat brain tissues were immersed in 4% PFA for 5 days at 4°C for postfixation. The tissue was then immersed in sucrose for 2 days in preparation for cryosectioning. The tissue was cut with a microtome (REM‐700; Yamato Kohki Industrial Co., Ltd., Saitama, Japan) to a thickness of 40 μm to create sections for observation. The sections were divided into three groups, so that approximately the same brain region was included. Using the first group of sections, mRFP1 fluorescence in the hypothalamus was observed and photographed using a fluorescence microscope (ECLIPSE Ti‐E; Nikon, Tokyo, Japan) with an RFP filter (Nikon). The obtained image was analyzed by an image analysis system (NIS‐Elements; Nikon), as described in our previous reports (Ueno et al., 2018). The PVN and SON in the hypothalamus were identified with reference to a rat atlas (Paxinos & Watson, 2013).

### 2.5 Percentage of Fos‐IR expression in the OXT‐mRFP1‐positive cells in the hypothalamus

A fluorescent protein was used to label c‐Fos protein using the immunohistochemical staining method described in previous reports (Motojima et al., 2016; Ueno et al., 2018, 2019). The second group of sections mentioned above was incubated for 5 days at 4°C with a primary anti‐Fos antibody solution (#sc‐52; rabbit, Santa Cruz Biotechnology, Dallas, TX, USA; 1:500 in phosphate‐buffered saline [PBS]). After washing for 20 min, the sections were treated with a secondary antibody solution for 2 hr (Alexa Flour 488 goat anti‐rabbit IgG; #A11035; Molecular Probes, Eugene, OR, USA; 1:1,000 in PBS).

The expression of Fos‐IR in cells labeled by green fluorescence was photographed by a fluorescence microscope using a green fluorescent protein (GFP) filter (Nikon). Then, the expression of OXT‐mRFP1 labeled by red fluorescence in the same section was also photographed, and these two images were merged using an image analysis system. The examination for the percentage of Fos‐IR expression in the OXT‐mRFP1‐positive cells enabled estimation of OXT neuronal activity in the mPVN, dpPVN, and SON.

### 2.6 Fos‐IR expression in the OVLT, MnPO, SFO, AP, NTS, and RVLM

Fos‐IR expression was also observed in the OVLT, MnPO SFO, AP, NTS, and RVLM, using the sections that verified Fos‐IR expression in the OXT‐mRFP1‐positive neurons in the PVN and SON. Each of the observed nuclei was identified by reference to the rat atlas (Paxinos & Watson, 2013). Next, the numbers of Fos‐IR‐expressing cells in the OVLT, MnPO, SFO, AP, NTS, and RVLM were determined manually.

### 2.7 In situ hybridization histochemistry for *mRFP1* and *OXT*

To measure the changes of *mRFP1* and *OXT* gene expressions in the PVN and SON for analysis by in situ hybridization histochemistry, transgenic rats were sacrificed by decapitation at 3 and 6 hr after administration of saline, HTN, and PEG at 10:00–12:00 (*n* = 6 in each group). In situ hybridization in this study was performed based on previous reports (Ueno et al., 2018, 2019; Ueta, Levy, et al., 1995). Thus, only a brief explanation of the procedure of in situ hybridization is provided in this article. Brain tissue was frozen immediately and carefully by crushed dry ice after decapitation. The tissues were cut with a microtome to a thickness of 12 μm to create sections for observation of in situ hybridization. As mentioned above, the nucleus to observe was identified by referring to the rat atlas (Paxinos & Watson, 2013).

The ^35^S‐labeled oligodeoxynucleotide probe and protocol for in situ hybridization histochemistry (ISH) in this experiment have been used many times before, and its reliability is considered guaranteed. The details of the probe sequence and the protocol for ISH have been described previously (Ueno et al., 2018, 2019; Ueta, Levy, et al., 1995). The hybridized sections were exposed to autoradiography films (Amersham Hyperfilm, Buckinghamshire, UK) for 3 days (*mRFP1*) and 6 hr (*OXT*). The gene expression in the obtained image was analyzed semiquantitatively using the ImageJ system (National Institutes of Health, Baltimore, MD, USA).

### 2.8 Measurement of plasma OXT levels

Plasma OXT levels were measured in rat blood samples taken at decapitation by a radioimmunoassay (RIA) method using specific anti‐OXT antisera (*n* = 6 in each group) (Onaka & Yagi, 1990). The intra‐/inter‐assay coefficients of variation for measuring plasma OXT levels were 4/10% with this method.

### 2.9 Statistical analysis

Data for the analysis of fluorescence intensity, immunohistochemistry, and in situ hybridization are presented as the mean percentages of the untreated group ± standard error of the mean (*SEM*). Results for different groups of transgenic rats were compared by one‐way ANOVA with the Bonferroni post hoc test for multiple comparisons, and they were considered significant if *p* < .05.

## 3 RESULTS

### 3.1 Hypothalamic mRFP1 fluorescence intensity

**FIGURE 1:**
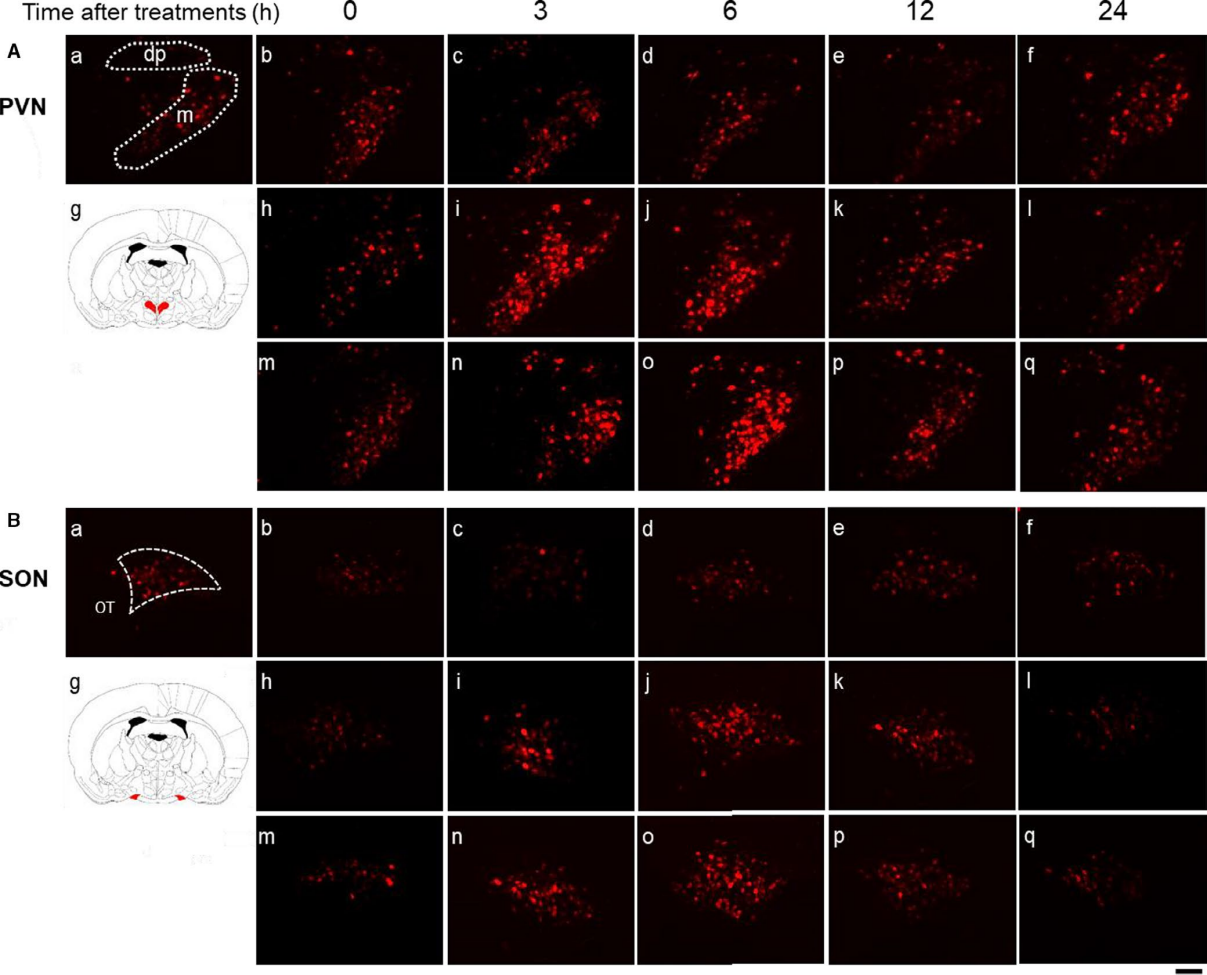
Representative images of OXT‐mRFP1 fluorescence after administration of saline, HTN, and PEG in the PVN (A) and SON (B). Each image was selected from untreated (A, B‐a), saline‐treated (A, B‐b‐f), HTN‐treated (A, B‐h‐l), and PEG‐treated (A, B‐m‐q) animals at 0 (b, h, m), 3 (c, i, n), 6 (d, j, o), 12 (e, k, p), and 24 (f, l, q) h after administration (each group at each time point, *n* = 6). A schematic anatomic representation of the nuclei (green shading) adapted from the rat brain atlas (A, B‐g). m: magnocellular division of the PVN, dp: dorsal division of the PVN. Black scale bar = 100 µm

**FIGURE 2:**
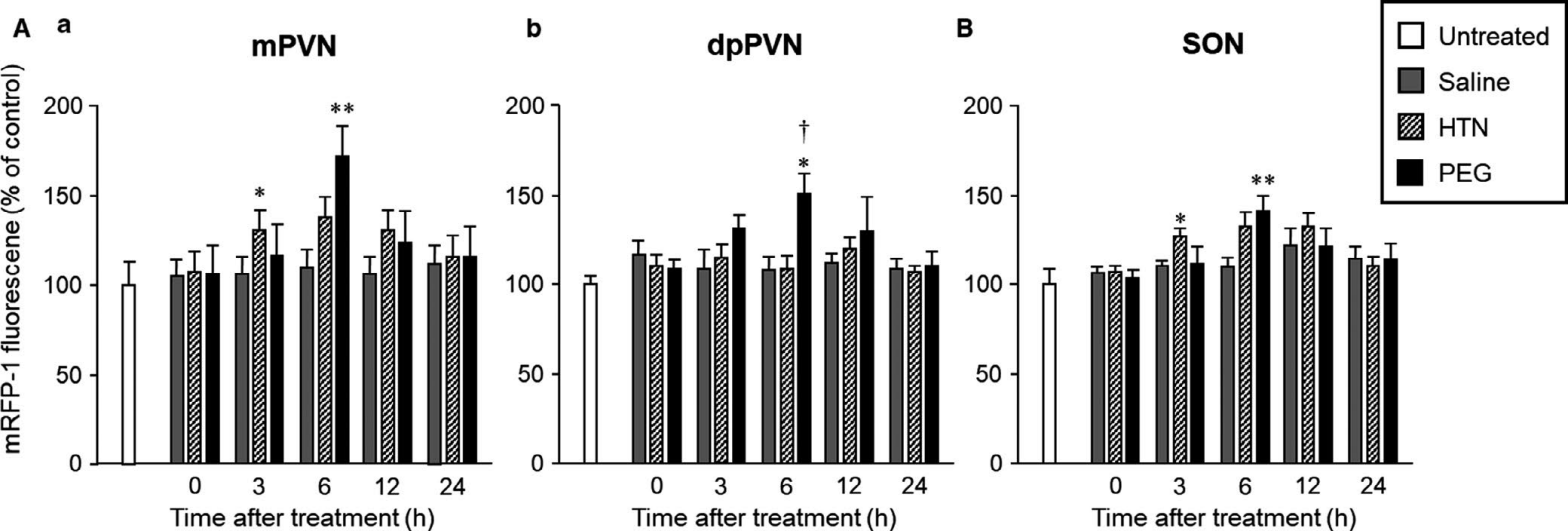
Analysis of OXT‐mRFP1 fluorescence intensity in the PVN (A‐a; m, b; dp) and SON (B) at 0, 3, 6, 12, and 24 hr after administration of saline, HTN, or PEG. Data are presented as means ± *SEM* (each group at each time point, *n* = 6). Significant if *p* < .05 on ANOVA. *p < .05, **p < .01 compared with the saline‐treated group. ^†^ *p* < .05, ^††^ *p* < .01 compared with the HTN‐treated group

Representative digital images of mRFP1 fluorescence in the PVN (Figure 1A) and SON (Figure 1B) in untreated (Figure 1A, B‐a), saline‐administered (Figure 1A,B‐b‐f), HTN‐administered (Figure 1A, B‐h‐l), and PEG‐administered (Figure 1A, B‐m‐q) rats at 0 hr (Figure 1A, B‐b, h, m), 3 hr (Figure 1A, B‐c, i, n), 6 hr (Figure 1A, B‐d, j, o), 12 hr (Figure 1A, B‐e, k, p), and 24 hr (Figure 1A, B‐f, l, q) after treatment are shown. Intense mRFP1 fluorescence could be observed in the PVN at 6 hr after PEG administration (Figure 1A‐o). The mRFP1 fluorescence intensity in the mPVN, dpPVN, and SON was significantly increased at 6 hr in the PEG‐administered group (Figure 2A, B). The mRFP1 fluorescence intensity at 3 hr in the mPVN and SON was significantly higher in the HTN‐treated group than in the saline‐treated group (Figure 2A‐a, B).

### 3.2 Fos‐IR expression in OXT‐mRFP1‐positive neurons in the hypothalamus

**FIGURE 3:**
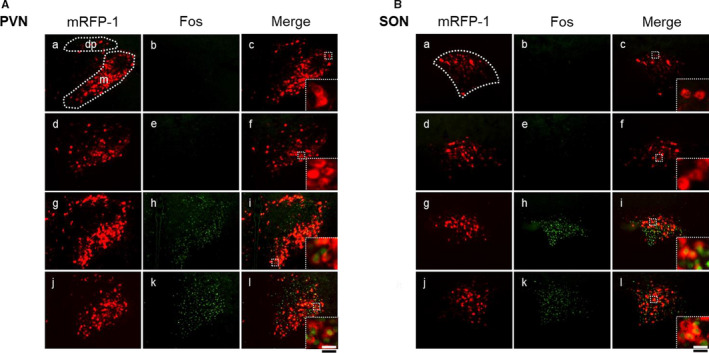
Representative images of OXT fluorescence and fluorescent immunohistochemistry for Fos‐IR expression in the PVN (A) and SON (B) in OXT mRFP transgenic rats 1.5 hr after administration of saline, HTN, or PEG. OXT mRFP‐positive neurons (appearing as red cytoplasmic cells) (A, B‐a, d, g, j), Fos‐IR‐positive nuclei (appearing as round‐shaped and green‐colored) (A, B‐b, e, h, k), and merged images (appearing as round‐shaped and red‐colored) (A, B, C‐c, f, i, l) in the PVN (A) and SON (B) of the untreated (A, B‐a, b, c), saline‐treated (A, B‐d, e, f), HTN‐treated (A, B‐g, h, i), and PEG‐treated groups (A, B‐j, k, l) (each group, *n* = 6) were selected. Black scale bar = 100 µm

**FIGURE 4:**
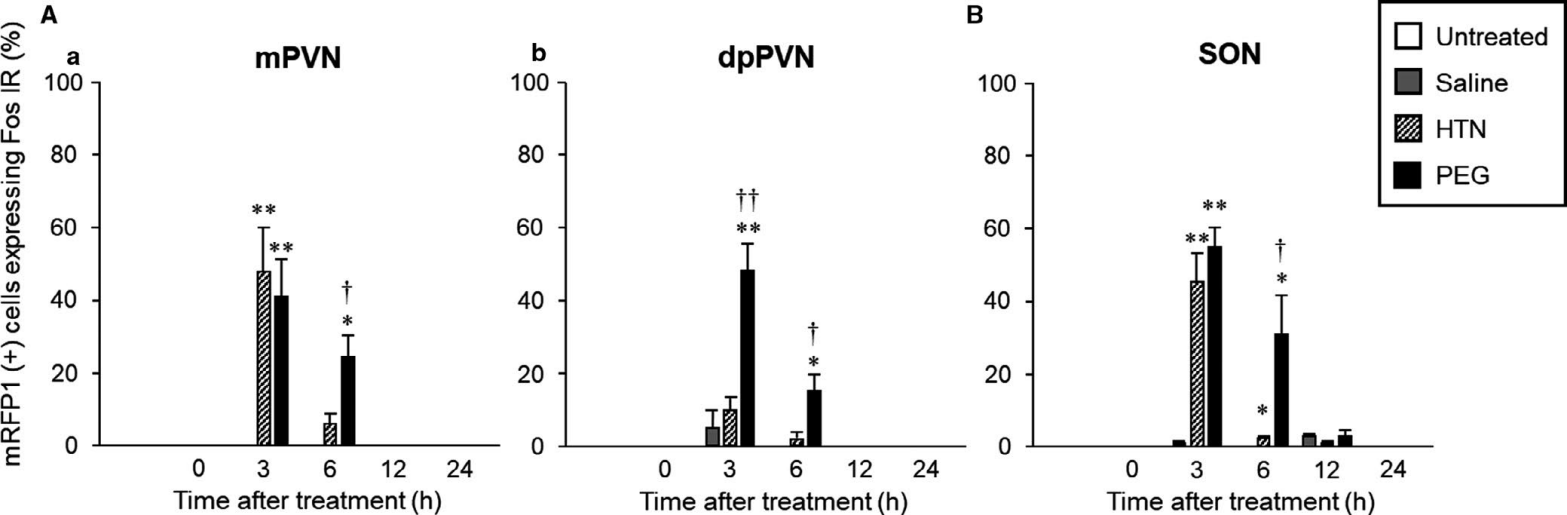
Analysis of the average percentage of mRFP1‐positive neurons expressing Fos‐IR in the PVN (A‐a; m, b; dp) and SON (B) at 0, 3, 6, 12, and 24 hr after administration of saline, HTN, or PEG. Data are presented as means ± *SEM* (each group at each time point, *n* = 6). Significant if *p* < .05 on ANOVA. *p < .05, **p < .01 compared with the saline‐treated group. ^†^ *p* < .05, ^††^ *p* < .01 compared with the HTN‐treated group

Representative digital images of OXT‐mRFP1‐positive neurons (Figure 3A, B‐a, d, g, j), Fos‐IR expression (Figure 3A, B‐b, e, h, k), and composite pictures (Figure 3A, B‐c, f, i, l) in the PVN (Figure 3A) and SON (Figure 3B) in untreated (Figure 3A, B‐a‐c), saline‐administered (Figure 3A, B‐d‐f), HTN‐administered (Figure 3A, B‐g‐i), and PEG‐administered (Figure 3A, B‐j‐l) rats at 3 hr after treatment are shown. The mRFP1‐positive neurons were confirmed as red cytoplasmic precipitates, and Fos‐IR‐expressing cells were identified by green nuclei. The percentage of Fos‐IR expression in mRFP1‐positive neurons in the mPVN, dpPVN, and SON at 3 and 6 hr after treatment in the PEG‐treated group was markedly increased compared with the saline‐treated group (Figure 4A, B). The percentage of Fos‐IR expression in mRFP1‐positive neurons in the mPVN and SON in the PEG‐treated group at 3 hr after treatment was markedly increased compared with the saline‐treated group (Figure 4A‐a, B).

### 3.3 Fos‐IR expression patterns in the OVLT, MnPO, SFO, AP, NTS, and RVLM

**FIGURE 5:**
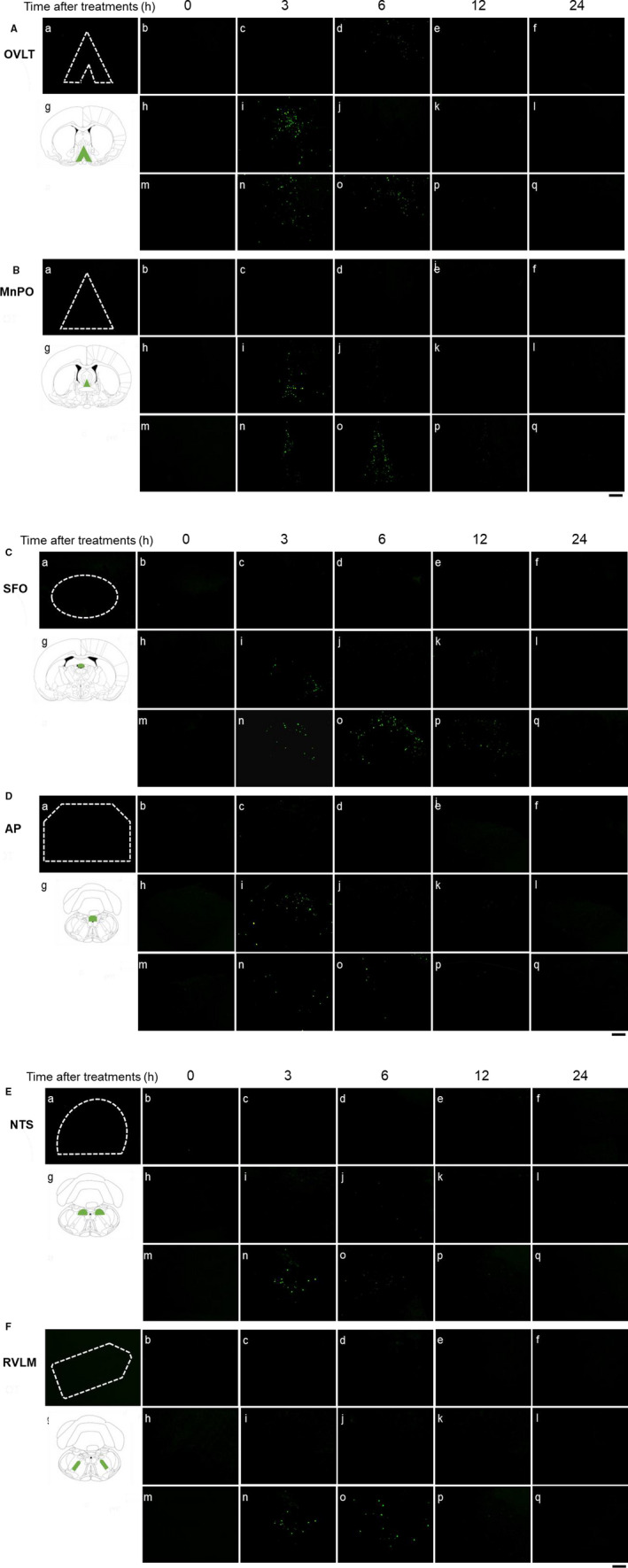
Representative images of fluorescent immunohistochemistry for Fos‐IR expression after administration of saline, HTN, or PEG in the OVLT (A), MnPO (B), SFO (C), AP (D), NTS (E), and RVLM (F). Each image was selected from untreated (A, B‐a), saline‐treated (A, B‐b‐f), HTN‐treated (A, B‐h‐l), and PEG‐treated (A, B‐m‐q) animals at 0 (b, h, m), 3 (c, i, n), 6 (d, j, o), 12 (e, k, p), and 24 (f, l, q) h after administration (each group at each time point, *n* = 6). A schematic anatomic representation of the nuclei (green shading) adapted from the rat brain atlas (A, B‐g). Black scale bar = 100 µm

**FIGURE 6:**
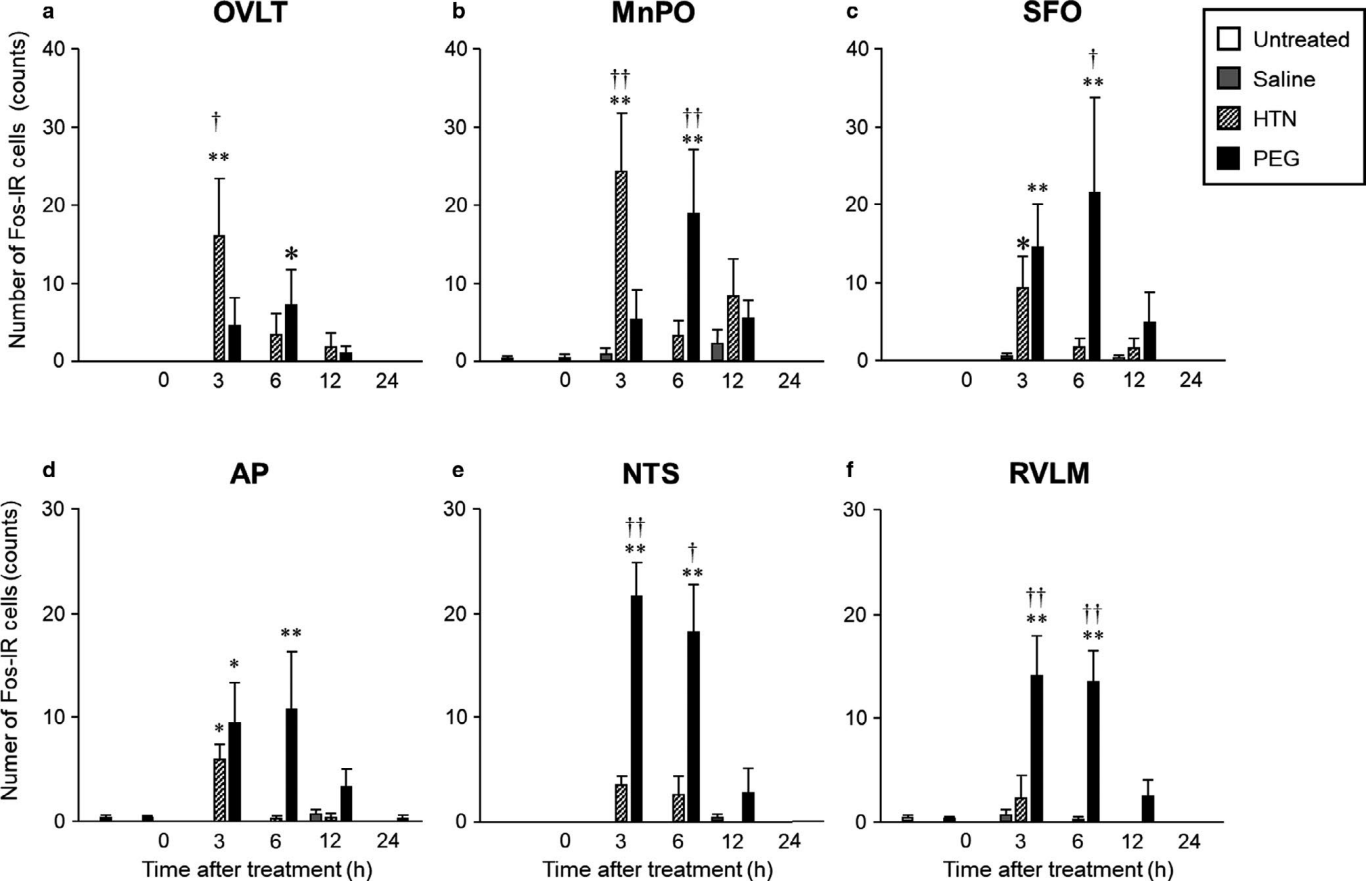
The numbers of Fos‐IR‐expressing cells after administration of saline, HTN, or PEG in the OVLT (A), MnPO (B), SFO (Fig. C), AP (D), NTS (E), and RVLM (F). Data are presented as means ± *SEM* (each group at each time point, *n* = 6). Significant if *p* < .05 on ANOVA. *p < .05, **p < .01 compared with the saline‐treated group. ^†^ *p* < .05, ^††^ *p* < .01 compared with the HTN‐treated group

**FIGURE 7:**
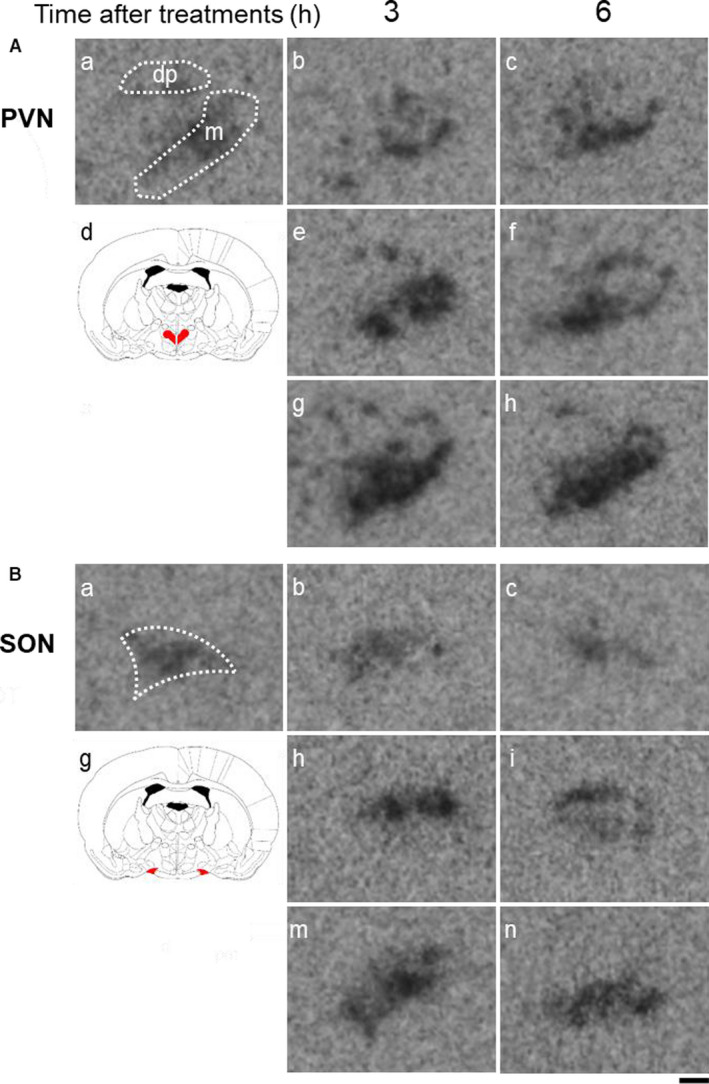
Representative images hybridized with a ^35^S‐labeled oligodeoxynucleotide probe complementary to *OXT* in the PVN (A) and SON (B) at 3 and 6 hr after administration of saline, HTN, or PEG. Each image was selected from untreated (A, B‐a), saline‐treated (A, B‐b, c), HTN‐treated (A, B‐e, f), and PEG‐treated (A, B‐g, h) animals at 3 (A, B‐b, e, g) and 6 (A, B‐c, f, h) h after administration (each group at each time point, *n* = 6). A schematic anatomic representation of the nuclei (red shading) adapted from the rat brain atlas (A, B‐d). m: magnocellular division of the PVN, dp: dorsal division of the PVN. Signal intensity ranges from high (black) to low (white)

**FIGURE 8:**
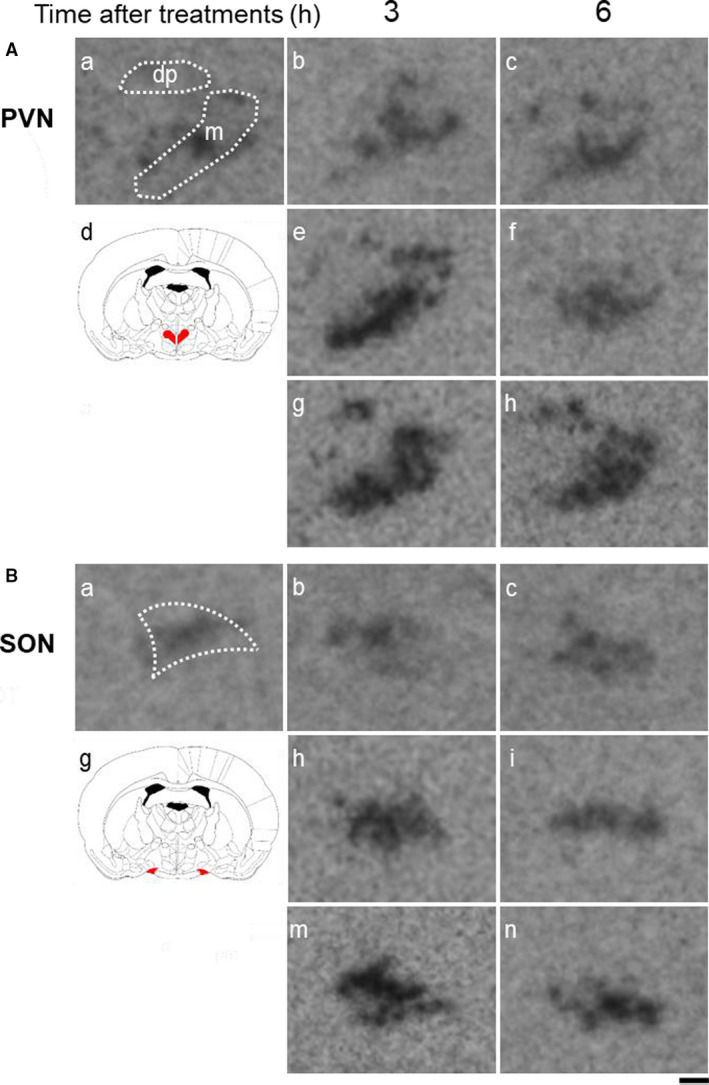
Representative images hybridized with a ^35^S‐labeled oligodeoxynucleotide probe complementary to *mRFP1* in the PVN (A) and SON (B) at 3 and 6 hr after administration of saline, HTN, or PEG. Each image was selected from untreated (A, B‐a), saline‐treated (A, B‐b, c), HTN‐treated (A, B‐e, f), and PEG‐treated (A, B‐g, h) animals at 3 (A, B‐b, e, g) and 6 (A, B‐c, f, h) h after administration (each group at each time point, *n* = 6). A schematic anatomic representation of the nuclei (red shading) adapted from the rat brain atlas (A, B‐d). m: magnocellular division of the PVN, dp: dorsal division of the PVN. Signal intensity ranges from high (black) to low (white)

**FIGURE 9:**
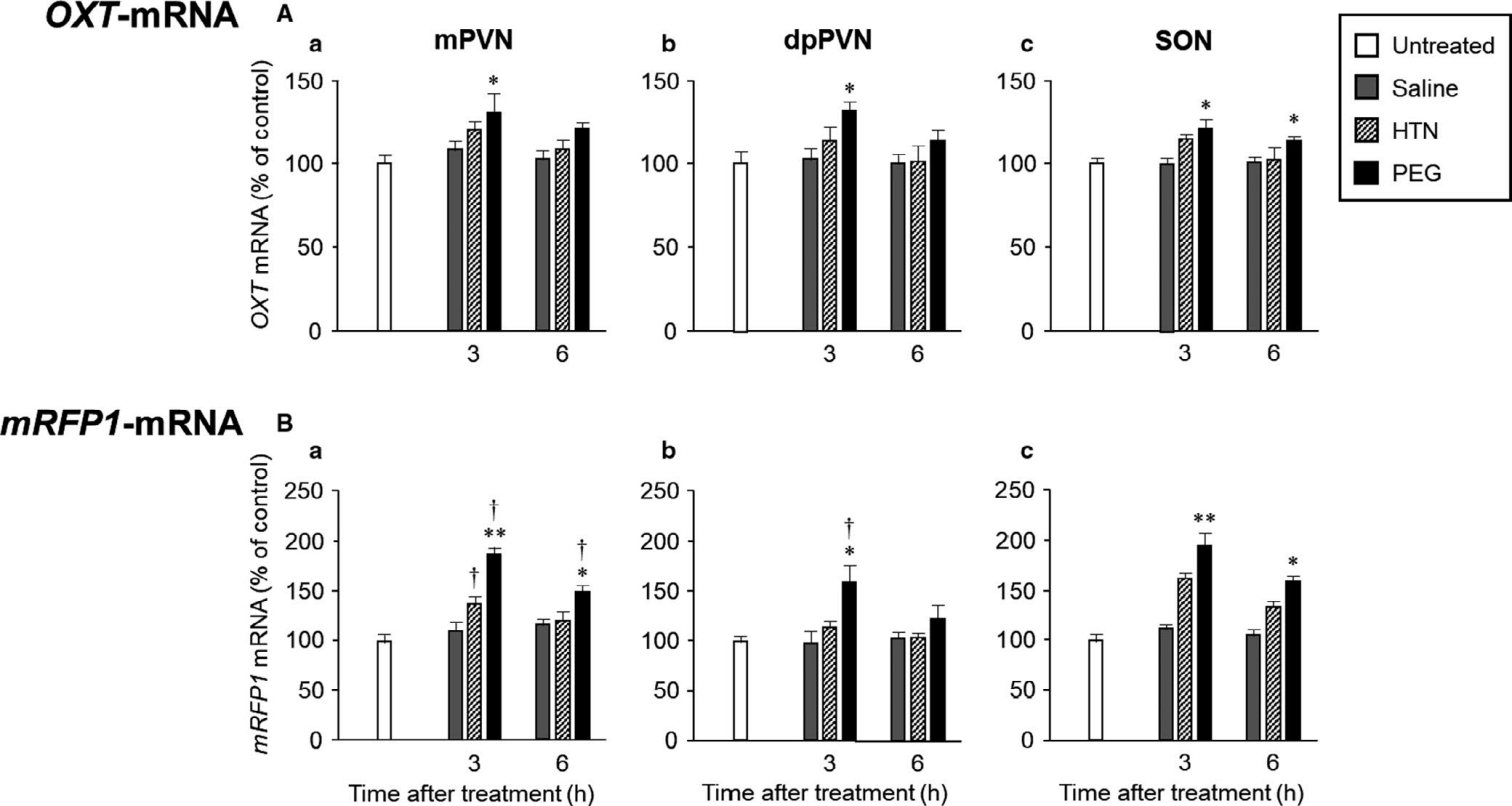
Effects of administration of saline, HTN, or PEG on the gene expressions of *OXT* (A) and *mRFP1* (B) in the mPVN (A, B‐a), dpPVN (A, B‐b), and SON (A, B‐c). Data are presented as means ± *SEM* (each group at each time point, *n* = 6). Significant if *p* < .05 on ANOVA. *p < .05, **p < .01 compared with the saline‐treated group. ^†^ *p* < .05, ^††^ *p* < .01 compared with the HTN‐treated group

**FIGURE 10:**
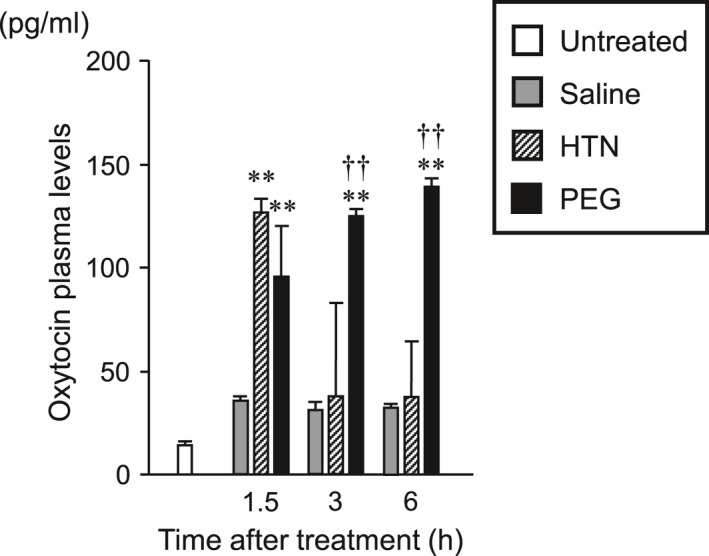
The plasma levels of OXT at 1.5, 3, and 6 hr after administration of saline, HTN, or PEG. Data are presented as means ± *SEM* (each group at each time point, *n* = 6). Significant if *p* < .05 on ANOVA. *p < .05, **p < .01 compared with the saline‐treated group. ^†^ *p* < .05, ^††^ *p* < .01 compared with the HTN‐treated group

**FIGURE 11:**
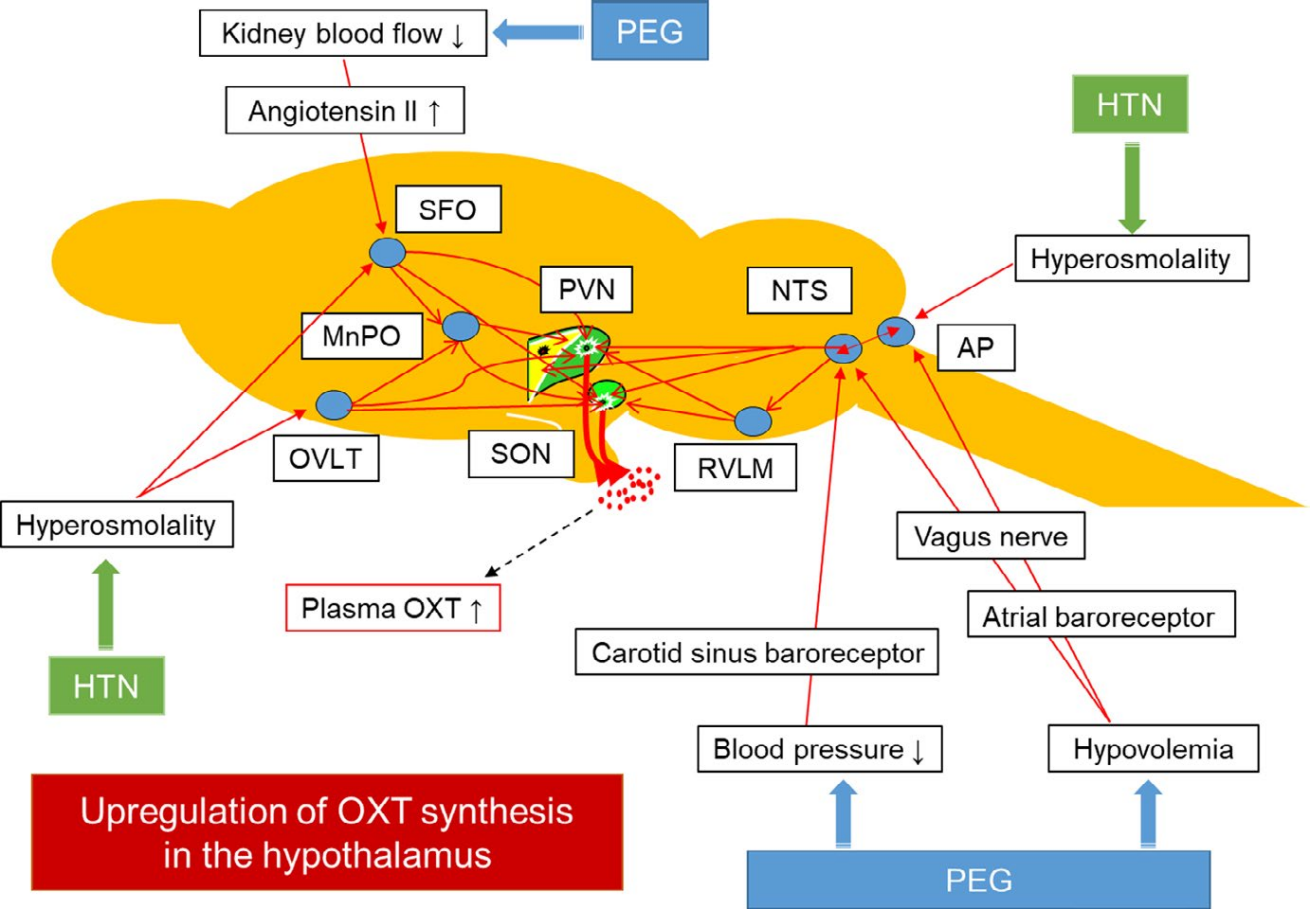
OXT neuronal circuit activation under osmotic challenge and acute hypovolemia. CVOs activation under hyperosmolality induced by HTN, and brainstem neurons and SFO activation under hypovolemia induced by PEG could upregulate OXT synthesis in the hypothalamus

Representative digital images of Fos‐IR expression in the OVLT (Figure 5A), MnPO (Figure 5B), SFO (Figure 5C), AP (Figure 5D), NTS (Figure 5E), and RVLM (Figure 5F) in untreated (Figures 5, 6, 7, 8, 9, 10, 11a), saline‐administered (Figures 5, 6, 7, 8, 9, 10, 11b‐f), HTN‐administered (Figure 5A, B‐h‐l), and PEG‐administered (Figure 5A, B‐m‐q) rats at 0 hr (Figure 5A, B‐b, h, m), 3 hr (Figure 5A, B‐c, i, n), 6 hr (Figure 5A, B‐d, j, o), 12 hr (Figure 5A, B‐e, k, p), and 24 hr (Figure 5A, B‐f, l, q) after treatment are shown. The numbers with Fos‐IR expression in the OVLT (Figure 6A), MnPO (Figure 6B), SFO (Figure 6C), AP (Figure 6D), NTS (Figure 6E), and RVLM (Figure 6F) at 3 and/or 6 hr were significantly higher in the PEG‐administered group than in the saline‐administered group. On the other hand, the numbers with Fos‐IR expression in the OVLT (Figure 6A), MnPO (Figure 6B), SFO (Figure 6C), and AP (Figure 6D) at 3 hr were significantly higher in the HTN‐administered group than in the saline‐administered group.

### 3.4 Gene expression of OXT in the hypothalamus

Representative images of *OXT* gene expression in the PVN (Figure 7A) and SON (Figure 7B) in untreated (Figure 7A, B‐a), saline‐administered (Figure 7A, B‐b, c), HTN‐administered (Figure 7A, B‐e, f) and PEG‐administered (Figure 7A, B‐g, h) rats at 3 and 6 hr after treatment are shown. The *OXT* expression levels in the mPVN and dpPVN at 3 and/or 6 hr were markedly higher in PEG‐administered than in saline‐administered rats (Figure 9A‐a, b).

### 3.5 Gene expression of mRFP1 in the hypothalamus

Representative images of *mRFP1* gene expression in the PVN (Figure 8A) and SON (Figure 8B) in untreated (Figure 8A, B‐a), saline‐administered (Figure 8A, B‐b, c), HTN‐administered (Figure 6A, B‐e, f), and PEG‐administered (Figure 8A, B‐g, h) rats at 3 and 6 hr after treatment are shown. The *mRFP1* expression levels in the mPVN at 3 and 6 hr were markedly higher in PEG‐treated rats than in saline‐treated rats (Figure 9B‐a). The *mRFP1* expression levels in the mPVN at 3 hr were significantly higher in HTN‐treated rats than in saline‐treated rats (Figure 9B‐a). The *mRFP1* expression levels in the dpPVN at 3 hr were markedly higher in PEG‐treated rats than in saline‐treated rats and HTN‐treated rats (Figure 9B‐b). The *mRFP1* expression levels in the SON at 3 and 6 hr were markedly higher in PEG‐treated rats and HTN‐treated rats than in saline‐treated rats (Figure 9B‐c).

### 3.6 Plasma OXT levels

Plasma OXT levels in untreated, saline‐administered, HTN‐administered, and PEG‐administered rats at 1.5, 3, and 6 hr after treatment are shown in Figure 10. Plasma OXT levels at 1.5 hr after treatment were significantly higher in HTN‐administered rats than in saline‐administered rats (Figure 10). Plasma OXT levels at 1.5, 3, and 6 hr after treatment were dramatically higher in PEG‐administered rats than in saline‐administered rats (Figure 10).

## 4 DISCUSSION

In this study, upregulation of hypothalamic OXT in HTN‐ and PEG‐treated rats was demonstrated using an OXT‐mRFP1 transgenic rat line. First, the fluorescence intensity of mRFP1, which is a surrogate marker for endogenous OXT, in the mPVN, dpPVN, and SON of HTN‐ and PEG‐treated rats was dramatically increased after HTN and PEG administration. Second, expression of Fos‐IR cells in the mRFP1‐positive cells was clearly increased in the mPVN, dpPVN, and SON of HTN‐ and PEG‐treated rats. In other words, OXT neuron activation was demonstrated in the mPVN, dpPVN, and SON in the HTN‐ and PEG‐treated rats. Third, the numbers of Fos‐IR‐expressing cells in the OVLT, MnPO, SFO, AP, NTS, and RVLM after HTN and PEG administration were significantly higher than in the saline‐treated rats. Next, *OXT* and *mRFP1* expression levels in the mPVN, dpPVN, and SON of HTN‐ and PEG‐treated rats were dramatically increased. Finally, the plasma OXT level was dramatically increased in HTN‐ and PEG‐treated rats. These results were consistent with previous reports that osmotic challenge and hypovolemia affect OXT neuron and plasma levels (Giovannelli et al., 1990; Landgraf et al., 1988; Shibuki et al., 1988). In this study, the change over time of hypothalamic OXT synthesis under osmotic challenge and hypovolemia was evaluated quantitatively by analyzing the mRFP1 fluorescence intensity and ISH. The findings obtained in this experiment were as follows. First, there was upregulation of OXT‐mRFP1 synthesis and OXT neuron activation in the dpPVN under hypovolemia. Second, there was upregulation of OXT‐mRFP1 synthesis and OXT neuron activation in the mPVN under hypovolemia and hyperosmolality. Third, this study suggested that hypothalamic OXT synthesis increased more significantly under hypovolemia than under hyperosmolality.

In the present study, OXT synthesis in the hypothalamus under osmotic challenge and acute hypovolemia was investigated in greater detail by analyzing the mRFP1 fluorescence intensity and transcription of mRFP1 gene expression. Our previous reports showed that changes in hypothalamic OXT‐mRFP1 levels are significantly exaggerated compared to changes in endogenous OXT levels, and thus the OXT‐mRFP1 transgenic rats are a useful animal model for capturing changes in OXT under physiological conditions (Katoh et al., 2014; Nishimura et al., 2019). Indeed, the mRFP1 fluorescence and *mRFP1* expression levels showed strong responses under both osmotic challenge and acute hypovolemia. Whereas endogenous *OXT* levels in HTN‐treated rats were not significantly different compared to saline‐treated rats, and *mRFP1* levels in HTN‐treated rats showed a significant increase compared to saline‐treated rats. This result indicates that mRFP1 is superior to endogenous OXT in its detection sensitivity, as reported previously.

The previous reports of the effects of change in osmolality and circulating blood volume on OXT dynamics were reviewed. Salt loading, which is a chronic hyperosmotic stress, has been reported to increase *OXT* expression and plasma OXT levels in the hypothalamus (Katoh et al., 2014; Lightman & Young, 1987; Ventura et al., 2005). Other previous reports showed that plasma OXT levels increased under acute hypovolemia induced by PEG administration or hemorrhage and osmotic challenge induced by HTN administration (Rosella‐Dampman et al., 1987; Stricker & Verbalis, 1986; Weitzman et al., 1978). These previous reports confirm the credibility of the present experimental results. However, measuring plasma OXT levels alone is not sufficient to accurately verify hypothalamic OXT dynamics for two main reasons. First, OXT has a short half‐life in circulating blood post‐secretion (3–4 minutes; Leng & Sabatier, 2016). Second, OXT synthesized in the dpPVN is not released into circulation. To solve these problems, we believe that it is useful to directly observe hypothalamic OXT synthesis using transgenic rats, as in the present study.

Many previous reports have examined the physiological reaction in the central nervous system under hypovolemia without osmotic changes caused by i.p. administration of PEG (Kondo et al., 2004; Somponpun & Sladek, 2004; Ueta et al., 1998). In the present study, upregulation of OXT‐mRFP1 synthesis was seen under HTN‐induced osmotic challenge and PEG‐induced hypovolemia. Upregulation of OXT‐mRFP1 under osmotic challenge was observed earlier than in hypovolemia. There are two hypotheses as to the mechanism of this phenomenon. First, based on the past reports, the increases in plasma osmolality caused by HTN administration are expected to occur faster than in the hypovolemia caused by PEG administration. Second, there might be differences in the sensitivity of detecting changes in plasma osmolality and circulating blood volume. It has been reported that AVP synthesis and release are increased by sensitively sensing an osmotic change of about 1%. In contrast to the osmotic change, it has been reported that a 10%–20% circulating volume reduction is necessary for upregulation of AVP synthesis and release (Arima et al., 1999; Dunn et al., 1973). As shown in the present experiment, if the circulatory control mechanisms of AVP and OXT are similar under hyperosmolality and hypovolemia, these differences in detecting changes in osmolality and circulatory blood volume might be similar, too. Onaka et al. reported that plasma OXT increased only at more severe hypovolemia compared with AVP (Onaka & Yagi, 1990). Furthermore, it has been reported that the response sensitivity of the AVP control mechanism to hypovolemia is lower than that to hyperosmolality, but its response is greater (Hayashi et al., 2006). In fact, OXT‐mRFP1 upregulation under hypovolemia was greater than under acute osmotic challenge in the present experiment.

It is difficult to detect the change of *OXT* under hyperosmolality. Few studies have focused on the changes of *OXT* gene expression under osmotic challenge or hypovolemia. Lightman reported no change in *OXT* expression in the PVN 4 hr after HTN administration (Lightman & Young, 1988). This report matched the present data completely. In the present study, upregulation of endogenous *OXT* under hyperosmolality was not found. Carter and Murphy reported that there were no changes in hypothalamic *OXT* and *AVP* expressions after HTN and PEG administration (Carter & Murphy, 1991). The following two points can be considered as the reasons why their experimental results were different from the present experimental results. First, they evaluated hypothalamic OXT and AVP syntheses by the Northern blot technique. Second, they performed measurements at only two time points immediately after administration (Arase et al., 2018; Berecek & Swords, 1990). To the best of our knowledge, ISH is a better experimental method than Northern blot because it enables visualization of localization and quantitative evaluation. In their experiment, *AVP* gene expression also did not increase under hyperosmolality. This result was inconsistent with many previous studies that reported upregulation of hypothalamic AVP synthesis under hyperosmolality (Arima et al., 1999; Kawasaki et al., 2005). There was no significant difference, but hypothalamic *OXT* levels after PEG administration showed an increasing tendency in their experiment. We thought that there was small but significant upregulation of OXT synthesis under hyperosmolality. However, it might be difficult to detect this change by Northern blot. We believed that we could demonstrate the upregulation of hypothalamic OXT synthesis under hyperosmolality and hypovolemia by analyzing the mRFP1 fluorescent intensity and *mRFP1* expression.

Endogenous OXT is known to have natriuretic effects (Forsling et al., 1994; Walter et al., 2000). With this in mind, one might hypothesize that upregulation of OXT synthesis and secretion under hypovolemia enhance diuretic effects and exacerbate dehydration. However, previous papers suggested that OXT increases renal blood flow, resulting in natriuresis (Walter et al., 2000). Therefore, we believe that upregulation of hypothalamic OXT in hypovolemia does not exacerbate dehydration, but it helps maintain renal blood flow and homeostasis. We hypothesized that maternal hypovolemia at birth upregulates hypothalamic OXT. The upregulation of hypothalamic OXT could cause postpartum physiologic diuresis and may prevent flaccid bleeding by contracting the uterus. OXT has been reported to increase blood pressure and heart rate, like AVP (Mack et al., 2002). This vasopressor effect enhanced by OXT upregulation under hypovolemia could contribute to maintaining homeostasis during maternal hypovolemia at birth. The OXT receptor is known to be expressed in various regions in the brain. OXT upregulated by hyperosmolality and hypovolemia could have a central effect on these brain regions. For example, Rinaman et al. have shown c‐Fos expression in the amygdala, where the OXT receptor is localized, after HTN administration (Onaka & Yagi, 1991). OXT may be involved as a neurotransmitter in activation of the amygdala after sodium overload. However, previous reports have shown that sodium‐sensitive channels reside in the amygdala (Matsumoto, Leng, & MacGregor, 2015). Another possibility was that sodium overload might directly activate the amygdala. This experiment failed to show how upregulated OXT affected some brain regions downstream of the hypothalamic OXT neural circuit. This topic remains for future research.

In previous reports, the visualization of c‐Fos protein expression with immunohistochemical staining showed neuronal activity in the central nervous system under various physiological conditions (Cunningham, Grindstaff, Grindstaff, & Sullivan, 2002; Morgan et al., 1987; Ueno et al., 2018). In the present study, activation of the hypothalamic OXT neurons labeled by fluorescent protein and central nervous system areas that interact neuronally with the hypothalamus and/or are involved in maintaining water and electrolyte homeostasis was demonstrated under an osmotic challenge and acute hypovolemia by examining Fos‐IR expression. Previous reports reported that hyperosmolality or hypovolemia activates these brain regions by different experimental methods, and the results are consistent with the results of the present experiment (Geerling & Loewy, 2007; Hamamura et al., 1992; Smith & Day, 1995; Xiong & Hatton, 1996; Yoshimura et al., 2013). Although these results are not new, we believe that it is important to have confirmed the activation of these brain regions in this experimental protocol. To the best of our knowledge, this is the first report that c‐Fos expression in the OXT neurons increased after PEG administration. It has been reported that excitatory and inhibitory neurons co‐exist in hypothalamic OXT neurons (Leng et al., 2001). Thus, it is not true that OXT synthesis is necessarily upregulated if c‐Fos expression is increased in OXT neurons. We believed that it was important to evaluate hypothalamic OXT synthesis directly by ISH, as in this experiment. Neuronal activation in the OVLT, MnPO, SFO, and AP was found after HTN administration. On the other hand, there was neuronal activation in the OVLT, MnPO, SFO, AP, NTS, and RVLM after PEG administration. In the CVOs—which contain the OVLT, SFO, and AP—the blood–brain barrier is not developed, and the areas are suitable for sensing humoral signals such as changes in plasma osmolality (Sladek & Johnson, 1983; Vivas et al., 1990). Most of these signals received at the OVLT and SFO are transmitted to the hypothalamus via the MnPO (Antunes‐Rodrigues et al., 2013). The hyperosmolality induced by HTN administration activated the OVLT, MnPO, SFO, and AP. On the other hand, the NTS and RVLM are reported to be the nuclei involved in regulating the activity of the autonomic nervous system (Bisset & Chowdrey, 1988; Ueta, Yamashita, et al., 1995). In a previous paper, Fos‐IR expression in the NTS and RVLM was reported under hypovolemia induced by water restriction (Ueta, Yamashita, et al., 1995; Yoshimura et al., 2013). The hypovolemia is detected at volume receptors in the cardiac atria, and this signal activates the sympathetic nervous system to maintain homeostasis. In the present report, PEG‐induced hypovolemia could activate the NTS and AP. In addition, we have two hypotheses for the mechanism by which Fos‐IR was expressed in the OVLT, MnPO, SFO, and AP after PEG administration. First, it has been reported that CVOs have many angiotensin II type 1 (AT1) receptors and regulate drinking behaviors (McKinley, Allen, Burns, Colvill, & Oldfield, 1998). Renal ischemia under hypovolemia after PEG administration could activate the renin‐angiotensin‐aldosterone system. Fos expression of CVOs has been confirmed in the above‐described dehydrated rats, and a similar mechanism could exist in both hypovolemic animal models (Ueta, Yamashita, et al., 1995; Yoshimura et al., 2013). The other hypothesis is that PEG absorbed into the body after i.p. retention might increase plasma oncotic osmolality. Previous reports showed that the OXT neurons in the hypothalamus received excitatory glutamic synaptic inputs from the OVLT, MnPO, and SFO (Boudaba & Tasker, 2006; Honda, Negoro, Dyball, Higuchi, & Takano, 1990; Sapru, 2013). The afferent excitatory noradrenergic neurons project from NTS to OXT neurons in the hypothalamus (Leng et al., 1995; Meddle et al., 2000). There is a neural interaction between OXT neurons in the hypothalamus and RVLM, and this neuronal circuit is involved in regulating the cardiovascular system (Koba et al., 2018; Mack et al., 2002). Onaka et al. reported that decreasing blood pressure increased plasma OXT, and that sino‐aortic denervation negated this effect (Onaka & Yagi, 1991). Given the above reasons, activation in the various nerve nuclei identified in the present experiment could be involved in the upregulation of hypothalamic OXT synthesis.

Interestingly, the present study showed that hypothalamic parvocellular OXT synthesis was upregulated only under acute hypovolemia, not osmotic challenge. Parvocellular OXT neurons are projected to the autonomic nervous system of the brainstem and spinal cord, where they are involved in regulating autonomic nervous system function (Jiang et al., 2018; Richard et al., 1991; Sofroniew, 1980). Fos expression in the NTS and RVLM indicated sympathetic nervous system activation in the brainstem under PEG‐induced hypovolemia in the present study. Therefore, upregulation of parvocellular OXT could contribute to control of the autonomic nervous system under hypovolemia.

In the present study, the upregulation of hypothalamic OXT synthesis under acute osmotic challenge and hypovolemia was clearly demonstrated by analyzing the transgenic rat line from various angles. These results suggest that activation of CVOs under hyperosmolality and activation of brainstem neurons and SFO under hypovolemia may upregulate hypothalamic OXT synthesis (Figure 11). Endogenous OXT, as well as endogenous AVP, may play important roles in maintaining fluid homeostasis in coping with hyperosmolality and hypovolemia. However, we should note that previous reports have shown that stimuli that cause strong OXT secretion in rats do not always elicit similar responses in primates (Verbalis et al., 1987). It remains a topic for further research to clarify whether hyperosmolality and hypovolemia upregulate hypothalamic OXT synthesis in primates, including humans.

## AUTHOR CONTRIBUTIONS

Conceived and designed the research: H.U., T.M., Y.O., and Y.U. Performed the research: H.U., K.S., K. B., K.T., H.N., K.N., S.S., M.Y., T.M., and T.O. Analyzed the data: H.U., Y.O., M.A., S.S., and Y.U. Interpreted results of experiments: H.U., T.O., Y.O., and Y.U. Prepared figures: H.U. Edited and revised the manuscript: H.U. and Y.U. All authors approved the final version of the manuscript and agreed to be accountable for all aspects of the work in ensuring that questions related to the accuracy are answered. All authors designated as authors qualify for authorship, and all those who qualify for authorship are listed.

### 1 DISCLOSURE

All authors declare that they have no financial relationships to disclose.
